# Efficacy and safety of ligation of intersphincteric fistula tract in the treatment of anal fistula: A protocol for systematic review and meta-analysis

**DOI:** 10.1097/MD.0000000000023700

**Published:** 2021-01-29

**Authors:** Jiaji Zhang, Xilu Hao, Yican Zhu, Ronggang Luan

**Affiliations:** Air Force Hospital of Eastern Theater Command, Nanjing, Jiangsu province, China.

**Keywords:** anal fistula, ligation of intersphincteric fistula tract, meta-analysis, protocol

## Abstract

**Background:**

Anal fistula is characterized with perianal cellulitis, anorectal pain, smelly or bloody drainage of pus, and difficulty in controlling bowel movements. Ligation of intersphincteric fistula tract (LIFT) technique is a recently developed approach against anal fistula, and it could alleviate the pain of the patient, with little postoperative trauma, which can greatly shorten the wound healing time and hospitalization stay. We conduct the meta-analysis and systematic review to systematically evaluate the clinical efficacy and safety of LIFT in the treatment of anal fistula.

**Methods:**

Randomized controlled trials of LIFT against anal fistula will be searched in several Chinese and English databases. Two reviewers will independently conduct the literature extraction and risk of bias assessment. Statistical analysis will be conducted in RevMan 5.3.

**Results and conclusions:**

The study will help to systematically evaluate the clinical efficacy and safety of LIFT in the treatment of anal fistula.

**OSF Registration number:**

DOI 10.17605/OSF.IO/T4FUH

## 1 Introduction

Anal fistula is an external abnormal anatomical connection between the epithelialized surface of the anal canal and the perianal skin, with the symptoms of perianal cellulitis, anorectal pain, smelly or bloody drainage of pus, and difficulty in controlling bowel movements in some cases.^[1,2]^ An anal fistula commonly occurs in people with a history of anal abscesses, in a prevalence of 0.01% to 0.02% in Europe,^[3]^ 0.018% in the United Kingdom.^[4]^ Surgery is the only treatment for anal fistula, and there are many described surgical techniques against it, such as anal fistula incision, cutting seton. In conventional surgeries, the internal sphincter, external sphincter, and part of the superficial part should be cut, which is frequently followed by a long and complicated postoperative course.^[5]^

Sphincter-preserving surgery is a new direction in the treatment of anal fistulas, which could cure anal fistulas while protecting the function of the anal orifice to the utmost extent.^[6]^ Ligation of intersphincteric fistula tract (LIFT) technique is a recently developed approach against anal fistula, involving the secure closure of the internal and external opening and removal of infected cryptoglandular tissue through the intersphincteric approach.^[7–9]^ It could alleviate the pain of the patient, with little postoperative trauma, which can greatly shorten the wound healing time and hospitalization stay.

However, the success rate of LIFT against anal fistula differs in different studies.^[7,10,11]^ Therefore, to systematically evaluate the clinical efficacy and safety of LIFT in the treatment of anal fistula, we conduct the meta-analysis and systematic review.

## 2 Methods

### 2.1 Study registration

The meta-analysis protocol has been drafted under the guidance of the preferred reporting items for systematic reviews and meta-analyses protocols (PRISMA-P),^[12]^ and it has been registered on open science framework (OSF) on November 4, 2020. (Registration number: DOI 10.17605/OSF.IO/T4FUH).

### 2.2 Ethics

Ethical approval is not required for no patient enrolled and personal information collected, and the data are all derived from published studies.

### 2.3 Inclusion criteria

#### 2.3.1 Type of studies

Randomized controlled trials (RCTs) of LIFT in the treatment of anal fistula reported in Chinese and English will be included.

#### 2.3.2 Type of participants

Participants with a diagnosis of anal fistula will be included, regardless of nationality, race, age, gender, and source.

#### 2.3.3 Type of interventions

The study focuses on RCTs of LIFT in the treatment of anal fistula, and the techniques of control group will not be limited.

#### 2.3.4 Type of outcome measures

Outcome measures include success rate of LIFT, operative time, estimated blood loss, postoperative length of hospital stay, and postoperative complications.

### 2.4 Exclusion criteria

(1) Studies without completely described outcomes of interest;
(2) Duplicated published literatures;
(3) Studies of unable to retrieve the related data from the literature or the authors;
(4) Literatures with inappropriate randomization.

### 2.5 Search strategy

Embase, Cochrane Library, PubMed, Medline, Chinese Biological and Medical database (CMB), China National Knowledge Infrastructure (CNKI), Chongqing VIP Chinese Science and Technology Periodical Database, and Wanfang database until November, 2020 will be searched, with the following words in various combinations: “ligation of intersphincteric fistula tract,” “anal fistulas,” “anal fistulae,” “fistula-in-ano.” The search strategy of PubMed is listed in Table 1.

**Table 1:** Retrieval strategy of PubMed.

Number	Search terms
#1	Anal fistula [Title/Abstract]
#2	Anal fistulae [Title/Abstract]
#3	Fistula-in-ano [Title/Abstract]
#4	Rectal fistula [MeSH Terms]
#5	FIA[Title/Abstract]
#6	#1 OR #2 OR #3 OR #4 OR#5
#7	Ligation of intersphincteric fistula tract [Title/Abstract]
#8	LIFT [Title/Abstract]
#9	Intersphincteric [Title/Abstract]
#10	#7 OR #8 OR #9
#17	#6 AND #10

### 2.6 Data extraction

**Figure 1:**
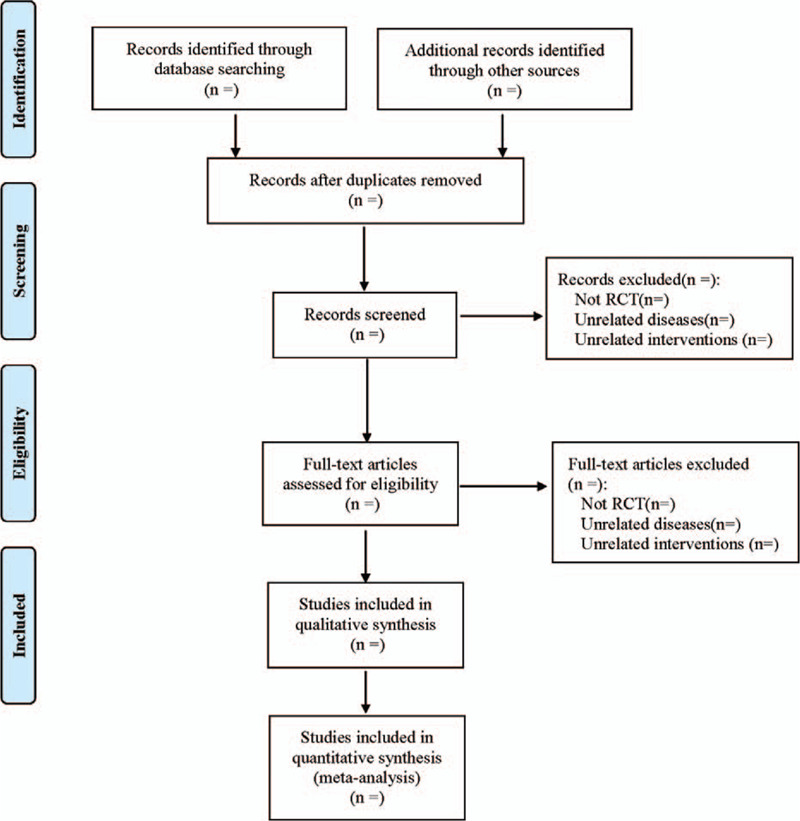
The process of literature screening.

The literature screening process is shown in Figure 1. Data extraction will be performed by 2 reviewers in Excel 2019 with an extraction table, including title, authors, journal, publication year, demographics, randomization, concealment, interventions, outcomes, adverse events. When comes across discrepancies, the senior author will be consulted to decide.

### 2.7 Risk of bias assessment

Two reviewers will independently evaluate the risk of bias in RCTs in accordance with the Cochrane Handbook of Systematic Reviewers, including random sequence generation, allocation concealment, blinding, incomplete outcomes, selective reporting, and other bias. The qualities of studies will be evaluated using the Newcastle-Ottawa Quality Assessment Scale. In case of disagreement, the author will be consulted.

### 2.8 Statistical analysis

#### 2.8.1 Data synthesis

Statistics will be analyzed by RevMan 5.3 (Cochrane Collaboration, Oxford, United Kingdom). The relative risk with the 95% confidence interval will be applied for dichotomous variables, and weighted Mean Difference with 95% confidence intervals for continuous variables. Heterogeneity test was evaluated with *Q* test and quantified with *I*^2^ statistic. It will be considered as statistical heterogeneity, and the random-effect model will be used for analysis without obvious clinical or methodological heterogeneity.

#### 2.8.2 Dealing with missing data

The corresponding author will be contacted by email to get the whole data when data is missing or incomplete in a study.

#### 2.8.3 Subgroup analysis

Subgroup analysis is conducted according to type of operation, such as sphincter anal fistula, intersphincter anal fistula, and superior sphincter anal fistula.

#### 2.8.4 Sensitivity analysis

A one-by-one elimination method will be adopted for sensitivity analysis to test the stability of meta-analysis results of indicators.

#### 2.8.5 Reporting bias

Funnel plot will be used to qualitatively detect publication bias if the included study is more than 10 for the major outcome indicators. Potential publication bias will be quantitatively assessed by Egger and Begg tests.

#### 2.8.6 Evidence quality evaluation

Evidence quality will be rated in high, moderate, low, and very low, by the Grading of Recommendations Assessment, Development, and Evaluation with bias risk, consistency, directness, precision, and publication bias.

## 3 Discussion

Surgery is supposed to be the only effective treatment for anal fistula. In the conventional techniques of incision or cutting seton, part of the anal sphincter needs to be cut or strangulated which will cause incontinence. The technique of LIFT was first raised by Rojanasakul et al^[13]^ in 2007, and it has been proved by many studies to be an effective and safe option against anal fistula.^[7,14,15]^ Therefore, in this study, we try to conduct this meta-analysis and systematic review to evaluate the efficacy and safety of LIFT against anal fistula. However, some limitation needs to be addressed. First, we only include studies in Chinese or English and it may result in certain selective bias. Second, there might be certain heterogeneity because of different techniques of LIFT performed in different RCTs.

## Author contributions

**Data curation:** Jiaji Zhang, Xilu Hao.

**Funding acquisition:** Ronggang Luan.

**Literature retrieval:** Jiaji Zhang and Xilu Hao.

**Software:** Yican Zhu.

**Supervision:** Yican Zhu.

**Writing – original draft:** Jiaji Zhang, Xilu Hao.

**Writing – review & editing:** Jiaji Zhang, Ronggang Luan.

## Abbreviations

Abbreviations: LIFT = Ligation of intersphincteric fistula tract, RCTs = randomized controlled trials.
